# Acute Oral Toxicity Evaluation of Almond Hull Powders in BALB/c Mice

**DOI:** 10.3390/foods12224111

**Published:** 2023-11-13

**Authors:** Juer Liu, Yuyang Yao, Yanling Cheng, Wei Hua, Xinyue Zhu, Qiming Miao, Guangwei Huang, Shengquan Mi, Roger Ruan

**Affiliations:** 1 Center for Biorefining and Department of Bioproducts and Biosystems Engineering, University of Minnesota, St. Paul, MI 55108, USA; liux3514@umn.edu (J.L.); cheng1012cn@aliyun.com (Y.C.); 2 Department of Food Science and Nutrition, University of Minnesota, St. Paul, MI 55108, USA; 3 Biochemical Engineering College, Beijing Union University, Beijing 100023, China; buuyaoyy@foxmail.com (Y.Y.); zss89661@163.com (X.Z.); miaoqiming1997@163.com (Q.M.); 4 Almond Board of California, Modesto, CA 95354, USA; ghuang@almondboard.com

**Keywords:** almond hull, acute oral toxicity, safety assessment, BALB/c mice, OECD 423

## Abstract

Almond hull, a substantial byproduct constituting more than half of almond fresh weight, has garnered recent attention due to its abundance in fiber and bioactive content. Despite this huge interest, data on its toxicity remain scarce. In line with the Organization for Economic Cooperation and Development (OECD) 423 guidelines, this study conducted an acute oral toxicity test using almond hull powders processed from three major almond varieties of Butte, Monterey, and Nonpareil on BALB/c female mice, administering dosages of 300 mg/kg body weight (bw), 2000 mg/kg bw, and 5000 mg/kg bw, with observations over a 14-day period. The results indicated that almond hull powders were non-toxic, aligning with the Globally Harmonized System’s classification. Administering up to 5000 mg/kg bw of all three varieties of almond hull powders (female BALB/c mice) and 10,000 mg/kg bw of Monterey almond hull powders (both female and male mice) induced no adverse effects in terms of mortality, body weight changes, food intake, organ to weight ratio, and clinical biochemistry. Additionally, histopathological examination revealed no organ abnormalities. This study demonstrates the non-toxic nature of almond hull as an edible food ingredient under experimental conditions, encouraging the further exploration of its potential for safe consumption and its health benefits.

## 1. Introduction

Almonds (*Prunus amygdalus*), belonging to the *Rosaceae* family, are one of the most popular tree nuts, accounting for the highest tree nut production worldwide [1]. California produces nearly 80% of the global almond output, yielding 1.32 million metric tons of almond kernels in the 2021/2022 crop year (Almond Almanac, 2022) [2]. Traditionally, almond kernels have been consumed as snacks and used as ingredients in processed foods, like bakery and confectionary products [3]. In recent years, they have gained traction as a constituent in dairy alternatives, gluten-free diets, and plant-based diets. This is attributed to their dense nutritional profile, which aids in reducing LDL cholesterol levels, promoting heart health, and supporting weight management [4].

Almond hulls, the thin mesocarp or green fruit flesh, represent the heaviest portion of the fruit and account for approximately 35–62% of the total almond fresh weight [5]. The annual production of almond hulls is around 1.6 times that of almond kernels [6]. Despite their significant presence in almond production, almond hulls have predominantly been utilized as a feed supplement for the dairy industry, and as soil amendments, resulted in an average price of approximately USD 110 per ton [7]. The inherent nature of almond hull imparts it with a rich assortment of nutrients and bioactive compounds, thereby endowing it with a protective capacity for the almond kernel. It has been reported that almond hulls have 25–33% fermentable sugars (glucose, fructose, sucrose) [8] and are also a valuable source of bioactive compounds, including three triterpenoids: betulinic, urosolic, and oleanolic acids, along with flavanol glycosides and phenolic acids [1]. Additionally, Takeoka and Dao [9] reported that almond hull extracts have a higher antioxidant activity than the equivalent concentration (10 µg/1 g of methyl linoleate) of alpha-tocopherol. It has also been proven that the phenolic-rich extracts of almond hull have a protective effect and can ameliorate oxidative stress in Caco-2 cells [10]. Furthermore, the maximum almond hull pectin (26.32% *w*/*w*) and almond hull phenolic compounds can be yielded at optimum point [11]. Almond hull also has a high total dietary fiber content, from approximately 46.3 to 57.9%, which can result in a good performance for their functionality, like water-holding capacity and emulsifying capacity [12]. 

A study conducted by Swanson and et al. indicated that the incorporation of almond hulls, up to 20%, in the diet of lactating dairy cows can lead to improved digestibility and an increase in milk fat percentage [13]. Also, almond hulls can be fed, up to 35%, to lactating goats without adversely affecting lactation [14]. One intriguing research study discovered that the inclusion of almond hulls at levels of 7.5% and 15% in the diet of laying hens did not have any significant effects on egg quality. However, it was observed that the hens in the hull diet group exhibited a reduction, in both fat and lean body mass, compared to the animals in the non-supplemented group [15]. Relevantly, it was found that pigs fed with the basal diet plus 15% of almond hulls ended up with 16% less body fat compared to the control group [16]. Upon the administration of almond hull powder in hyperlipidemic rats, the levels of cholesterol and triglycerides significantly decreased as the antioxidant capacity of plasma increased [17]. This may suggest a potential application of almond hulls as a dietary supplement for weight management in humans, though further research is needed. In a recent study conducted by Kahloui and et al. [18], it was reported that using almond hulls in bread production increased the fiber content, polyphenol content, and antioxidant activity. Breads containing mature almond hulls had the highest levels of fiber and sugars, mainly glucose. Consumer evaluation has shown that breads with 8% almond hull powder received the highest response in terms of overall consumer preference.

Despite the potential diverse applications for almond hulls, it is unfortunate that there are no current references available in the literature regarding their direct use for human food consumption, apart from unripen whole almond fruits (green almonds). Moreover, safety studies specifically focused on almond hull are conspicuously absent from the current body of research. Consequently, there arises an urgent need for comprehensive research aimed at assessing any potential safety concerns associated with the incorporation of almond hulls as a novel food ingredient. Therefore, the undertaking of this acute oral toxicity study holds significance for systematically evaluating any potential acute oral safety issues associated with almond hull by following the Organization for Economic Cooperation and Development (OECD) guidelines. Firstly, the findings of this study are expected to yield crucial insights and provide a foundational understanding regarding the safety profile of almond hulls for human consumption, which is vital for assessing the feasibility and safety of incorporating almond hulls into various and nutraceutical products, paving the way for the further safety assessments and sustainable utilization of almond hulls in the food industry. Secondly, the outcome of this research not only supports the ongoing research but also encourages future exploration and innovation in utilizing almond hulls within human food and nutraceutical products. Ultimately, this research contributes to the broader goal of enhancing food sustainability and diversifying consumer dietary choices through the utilization of this natural resource. 

## 2. Materials and Methods

### 2.1. Preparation of Almond Hull Powders 

Three types of almond hulls, namely Monterey (MT), Butte (BT), and Nonpareil (NP), harvested in 2021, were obtained from the Harris Woolf Almonds, located in Coalinga, CA, USA. The received raw hull samples were stored in a freezer at −20 °C until further processing. To prepare the samples, any undesirable materials such as shells, sticks, nuts, and stalks were removed, and the raw almond hulls were rinsed twice using cold tap water. Subsequently, the hulls were dried in a conventional oven at 60 °C for 48 h. After drying, the hulls were subjected to two rounds of grinding using a Wiley mill (3379-K05, Thomas Scientific, Chadds Fort, NJ, USA) with a 1 mm screen to achieve fine particles. The resulting fine particles were then sifted through a 100 mesh (149 μm) laboratory sieve after the milling process. The sample was sealed and refrigerated until use.

### 2.2. Assessment of Almond Hull Powders 

The fine MT powders of almond hull were evaluated for microbial, heavy metal, pesticide residue, and mycotoxins to confirm that their concentrations were all lower than the standard limit for each of these contaminants. There are no set industry standards or recommendations for the presence and levels of these microorganisms in almond hulls. However, according to the FDA 172.898-CFR-Code of Federal Regulations Title 21 [19], to achieve the viable microbial content of bakers yest glycan as a finished ingredient, it should have less than 10,000 CFU/gram by aerobic plate count, less than 10 CFU/gram for yeasts and molds, negative for *Salmonella*, *E. coli*, coagulase-positive *Staphylococci*, *Clostridium perfringens*, *Clostridium botulinum*, or any other recognized microbial pathogen or any harmful microbial toxin. The maximum residual levels (MRLs) for pesticide residues in almon hulls were acquired from the USDA MRL Database. Then, the almond hull powders were considered to be safe, before proceeding to the following studies, according to the results in Table 1.

### 2.3. Animals and Study Procedures

BALB/c mice of Specific Pathogen-Free (SPF) grade were purchased from Beijing Mei Lvzhou Biology Science and Technology Co., Ltd. (Beijing, China) (Certificate: SCXK2019-0008), weighing 18~22 g and being aged around 10 weeks. These animals were housed in appropriately sized polycarbonate cages with regular ventilation in an environmentally controlled room with a 12 h daily light and dark cycle, room temperature of 22 ± 2 °C, and relative humidity of 40~60%. Cage padding was replaced every three days. A pelleted diet (Beijing HFK Bioscience, Beijing, China) and sterilized drinking water were provided ad libitum. Following a 5-day quarantine and acclimation period, mice were randomly assigned to the control and treatment groups. This animal study has been approved by the Ethics Committee, Health Food Function Testing Center of Arts and Science College, Beijing Union University, China (Approval Code: No. 20220901). This study was conducted in accordance with the U.S. FDA Good Laboratory Practice (GLP) regulations, issued under Part 58. Title 21. Code of Federal Regulations. 

### 2.4. Acute Toxicity Assay 

**Figure 1:**
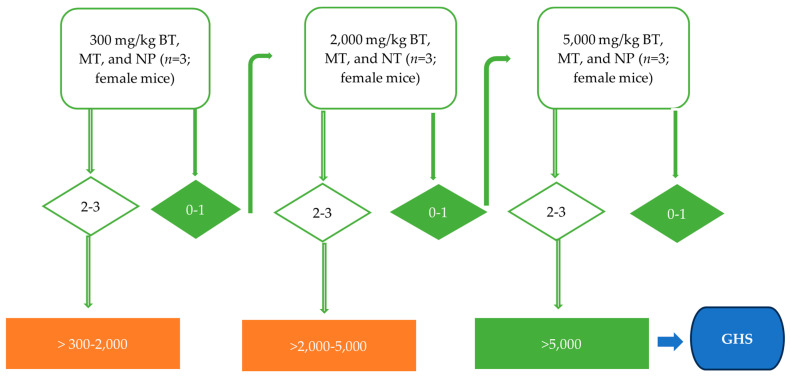
The test procedure (adapted from OECD guideline 423) shows the process for the LD_50_ cut-off value determination for almond hull powders. As per the guideline, if there is no information on a substance to be tested, for animal welfare reasons, it is recommended to use the 300 mg/kg body weight as a starting dosage. The acute toxicity study follows the stepwise procedure with the use of 3 animals of a single sex per step. If one or no mice die, a higher fixed dose level is used in the next step until the appropriate Globally Harmonized System (GHS) category is defined. Green arrows indicate the test procedure in our study. Abbreviations of almond hull powder: BT—Butte, MT—Monterey, NP—Nonpareil.

In accordance with the OECD Test Guidelines 420: Acute Oral Toxicity-Fixed Dose Procedure [20] and OECD Test Guidelines 423: Acute Oral Toxicity—Acute Toxic Class Method [21], the mice were kept without food for 10 h prior to dosing but had access to water ad libitum. The test articles (BT, MT, NP) were dissolved in 0.2% carboxymethyl cellulose (CMC) and sterile water (133.33 mg/mL); the mice in the control group in the follow-up studies were administered with 0.2% CMC at an equal volume. A total of 27 female mice were randomly assigned to the study according to the suggestion of the OECD 423 due to the higher sensitivity of females to the tests. The test articles were administered at doses of 300, 2000, and 5000 mg/kg body weight (bw) (*n* = 3). The gavage volume of 1 mL/100 g body weight using stomach tubes was adjusted according to the weight of each mouse. The animals were closely observed individually for the first 30 min for any signs of acute toxicity and behavioral changes, then for 4 h, and then at least once daily for 14 days. Food was provided after 1–2 h of dosing; since there is no information on almond hull to be tested before, for animal welfare reasons, the starting dose of 300 mg/kg was used, three animals were used for each step, and a 3-day interval was used to allow for observing the delayed toxicity before administering the next dose level. The flow charts describe the detailed experiment treatment schedule listed in Figure 1.

The body weight of an individual mouse was measured on the day of delivery, first treatment day, and once per 2~3 days thereafter until the end of the study period. All animals were monitored for clinical signs, including behavior, fur condition, eyes and mucous membranes, respiration, autonomic and central nervous systems, urine and fecal excretion, conditions of body orifices, behavior patterns, and any signs of illness. At the end of the 14-day observation period, all animals were weighted and euthanized by CO_2_ asphyxia, followed by execution with cervical dislocation. The blood samples were collected by cardiac puncture under anesthesia with isoflurane, and serum was separated for biochemical and hematological evaluations. Comprehensive necropsies were conducted on all subjects, involving meticulous excision, weighing, and fixation in 10% formalin of tissues and organs within the abdominal, thoracic, and cranial cavities. These specimens were meticulously prepared to facilitate subsequent histopathological assessments.

### 2.5. Biochemical Analysis

Blood samples were collected on the 14th day of the study after the animals were anesthetized. The EDTA-coated vials were used for the collection of serum samples. Total protein (TP), albumin (ALB), creatinine (CREA), alanine aminotransferase (ALT), aspartate aminotransferase (AST), and alkaline phosphatase (ALP) were measured using commercial reagent kits (Biosino Bio-technology, Beijing, China).

### 2.6. Histopathologic Study

The vital organs isolated from the sacrificed mice were then processed and embedded in paraffin wax. Random tissue sections were made at 5 mm increments of all the organs and then stained with hematoxylin and eosin. The slides were observed under an upright microscope (Nikon, Tokyo, Japan), and the magnified images of the tissue structures were captured for further study. 

### 2.7. Further Assessment of the Temporal Variation in Hepatic and Renal Function 

To further investigate the dynamic changes in the hepatic and renal function of the mice, a follow-up study was conducted using MT-administered mice with a specific focus on the hepatic function alterations at 24 h, 48 h, and 72 h intervals. Subsequently, an acute oral toxicity test was performed again followed by the main study, wherein the tested mice were administered MT at a dosage of 10,000 mg/kg of body weight, and the temporal variations in the hepatic and renal function were observed at 24 h, 48 h, 72 h, and 7 days post-administration. To comprehensively evaluate the safety profile of the test substance, a group of BALB/C male mice of the same standards of the original test animals were included in the study, each consisting of 6 individuals. Serum samples were collected post-test substance administration at specified time points to measure the hepatic and renal function markers. An additional group (*n* = 6) of male mice was designated as the control and received 0.2% carboxymethyl cellulose (CMC). Concurrently, a control group of BALB/C female mice (*n* = 6) and another group of BALB/C female mice were exposed to a 10,000 mg/kg dose of MT, facilitating comparative analysis. 

### 2.8. Statistical Analysis

SPSS 22.0 software and Graph Pad Prism 8.0 software were used to analyze body weights, food consumption, organ to body weight index, and biochemical analysis, followed by testing for normality and homogeneity of variances. Statistical analysis of the experimental results was presented as mean ± SD, and the statistical significance between the groups was analyzed by means of a one-way ANOVA and two-way ANOVA, followed by Tukey’s multiple comparison test. A *p* ≤ 0.05 was considered statistically significant. 

## 3. Results

### 3.1. Observations of Behavior Pattern 

All the animals were observed independently after dosing test articles 300–5000 mg/kg bw and showed no significant changes in skin condition, respiratory, circulatory, or behavior pattern. No mortality or abnormal clinical signs of the animals were observed during the experimental period.

### 3.2. Body Weight and Food Intake

**Figure 2:**
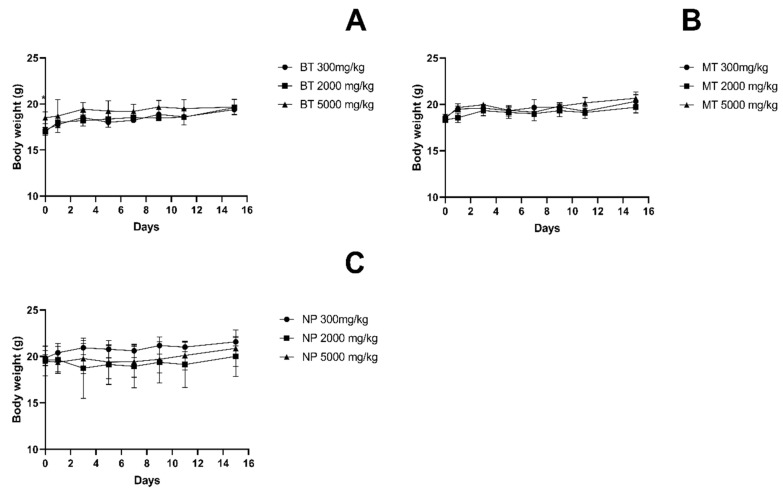
Effect of administration of almond hull powders on body weight in mice. (**A**) BT-treated mouse body weight. (**B**) MT-treated mouse body weight. (**C**) NP-treated mouse body weight. All data are reported as the mean ± SD for n = 3 per group. Two-way ANOVA, followed by Tukey’s test. * Significantly different, *p* < 0.05 between 2000 mg/kg and 5000 mg/kg. Abbreviations of almond hull powder: BT—Butte, MT—Monterey, NP—Nonpareil.

**Figure 3:**
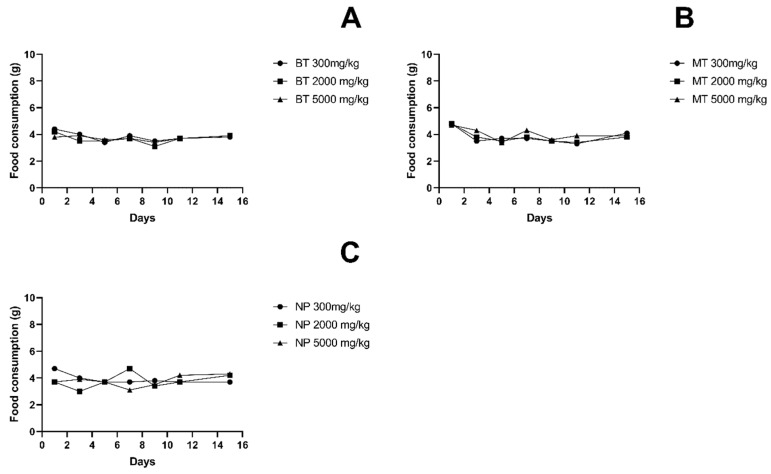
Effect of administration of almond hull powders on food consumption in mice. (**A**) BT-treated mouse body weight. (**B**) MT-treated mouse body weight. (**C**) NP-treated mouse body weight. Data expressed as mean for n = 3 per group. Abbreviations of almond hull powder: BT—Butte, MT—Monterey, NP—Nonpareil.

The body weights of the test animals of all treated groups increased gradually throughout the study, despite the varieties of almond hull and dosage, as shown in Figure 2. During the 14-day acute oral toxicity study, the results in Figure 3 show that there was no significant change in the food intake of the mice in all groups. 

### 3.3. Organ to Body Weight Index 

The major organs, including the heart, liver, spleen, stomach, kidney, lung, and thymus gland, from the mice were harvested and weighted, and then the organ to body weight index was calculated. Table 2 shows no statistically significant difference in the ratio of the heart, liver, spleen, stomach, kidney, and lung weight to body weight (bw) among the groups. However, the groups of mice administered BT almond hulls and NP almond hulls, at a dosage of 300 mg/kg bw, had a significant difference (*p* = 0.035) in the thymus gland to body weight index.

### 3.4. Biochemical Analysis

As shown in Table 3, there were no statistically significant differences in the creatinine levels observed across all the test groups. Regarding liver function tests, the levels of total protein, alkaline phosphatase, and albumin remained consistent among all the treated groups, showing no statistically significant changes among the nine test groups. The observed variations in alanine transaminase (ALT) levels within the NP-300 mg/kg bw and 5000 mg/kg bw-treated groups (*p* = 0.012) were deemed incidental and not attributed to the test article. Subsequently, a follow-up biochemical analysis was conducted to assess renal and liver function, along with a histopathological analysis.

### 3.5. Histopathological Analysis 

**Figure 4:**
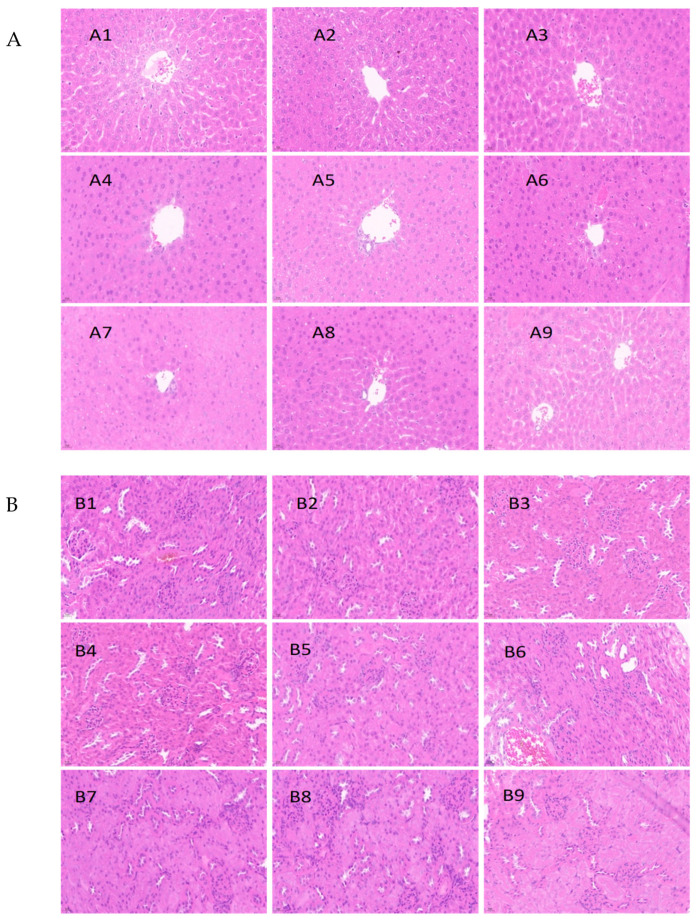

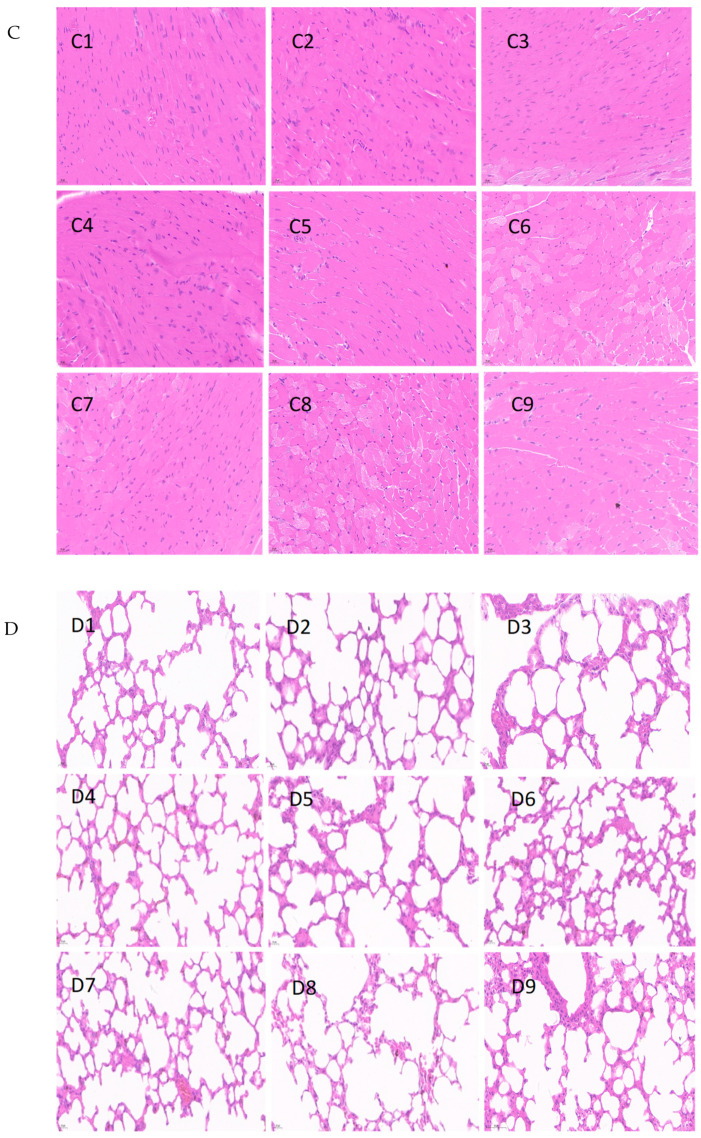

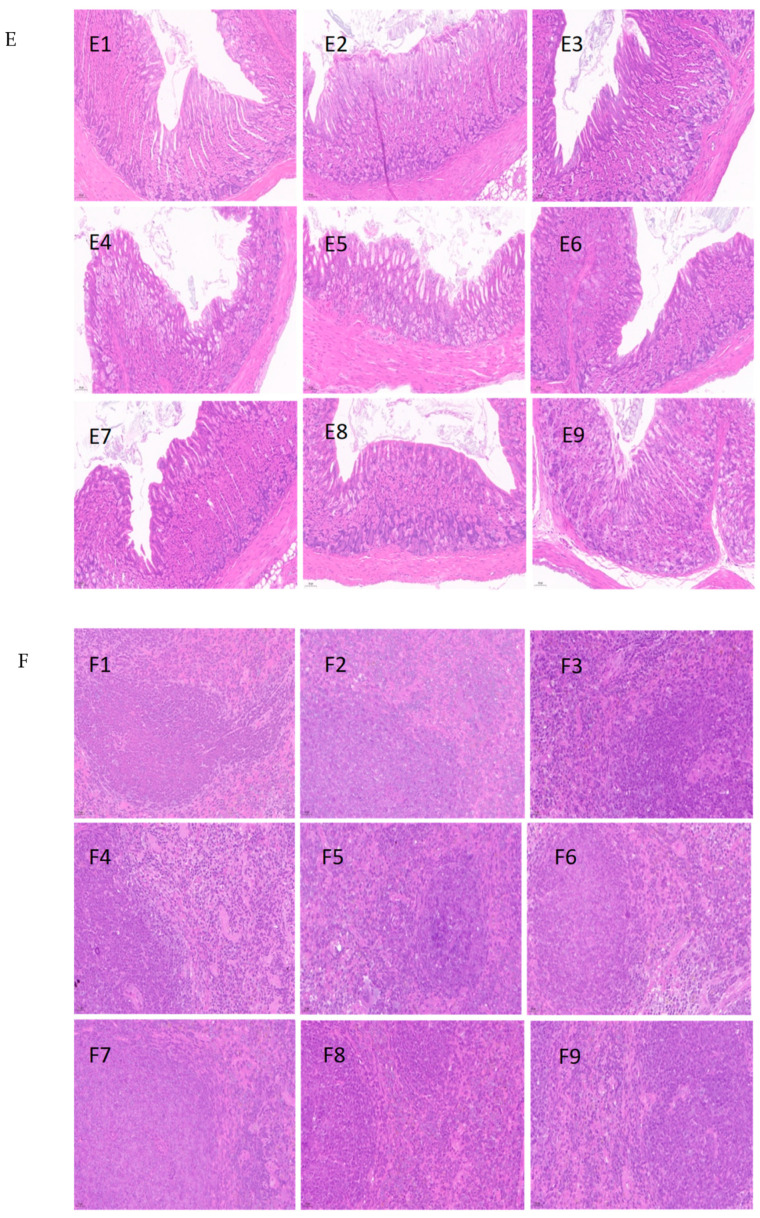
Histological examinations include liver (**A**), kidney (**B**), heart (**C**), lungs (**D**), stomach (**E**), and spleen (**F**) sections. 1: BT—300 mg/kg; 2: BT—2000 mg/kg; 3: BT—5000 mg/kg; 4: MT—300 mg/kg; 5: MT—2000 mg/kg; 6: MT—5000 mg/kg; 7: NP—300 mg/kg; 8: NP—2000 mg/kg; 9: NP—5000 mg/kg. (Magnification: ×40). Abbreviations of almond hull powder: BT—Butte, MT—Monterey, NP—Nonpareil.

The macroscopic examination of the organs of the animals treated with almond hull appeared to be in normal shape and color. Autopsies at the end of the experimental period revealed no apparent changes in the liver, kidney, lungs, heart, and spleen organs of the treated mice in the histopathology analysis. As shown in Figure 4A, the portal triad and central vein can be easily seen in the livers of treated mice, and there has been no indication of any patchy necrosis or hemorrhage. It can be observed from Figure 4B that the Bowman’s capsule, glomerulus, and renal corpuscle show a normal structure. Figure 4C indicates the normal appearance of the myocardial fibers of the treated mice; the lungs of the treated mice were considered to be healthy with the apparent alveoli and bronchiole (Figure 4D), no significant alterations were seen in the gastric mucosa in the treated mice’s stomachs (Figure 4E), and the connective tissue present within the spleen as the trabeculae that carry the arteries, veins, and nerves appeared to be of a normal status (Figure 4F). The histopathological examination revealed that none of the organs from the treated mice showed any alteration in cell structure or any unfavorable effects using multiple magnification power. The structure or coordination of the cells in the extract-treated organs were compared similarly to normal organs.

### 3.6. Further Assessment of the Temporal Variation in Hepatic and Renal Function

#### 3.6.1. Hepatic and Renal Function Biomarkers

**Figure 5:**
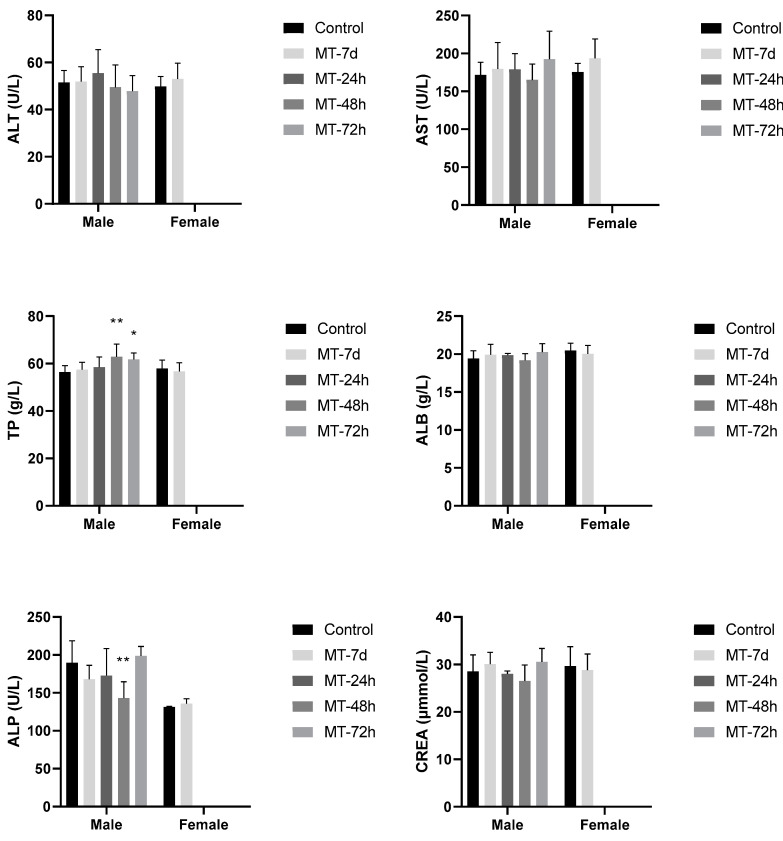
Effect of administration of MT—10,000 mg/kg almond hull powders on hepatic and renal function tests in male and female mice. Data expressed as mean ± SD. N = 6. One way ANOVA followed by Tukey test was used. * *p* < 0.05, ** *p* < 0.01. MT—Monterey almond hull powders.

No significant change in the serum creatinine level was observed compared to the control group (Figure 5). There were statistically significant differences in the TP levels at 48 h (*p* < 0.01) and 72 h (*p* < 0.05) in comparison to the male control group. Nonetheless, no such statistically significant difference was observed at 24 h and 7 d; this variation may be due to the increased food intake, resulting in higher TP levels. There was also a statistically significant difference in the ALP levels at 48 h (*p* < 0.01), which were lower than the value of the control group, while no statistically significant differences were observed in other groups. This variation may be due to random errors, dietary, and other non-test-article-related factors. No statistically significant differences in the ALT, AST, or ALB levels were observed. Based on the above data, no adverse reactions were observed. 

#### 3.6.2. Histological Evaluation of Liver and Kidney Tissue Sections 

**Figure 6:**
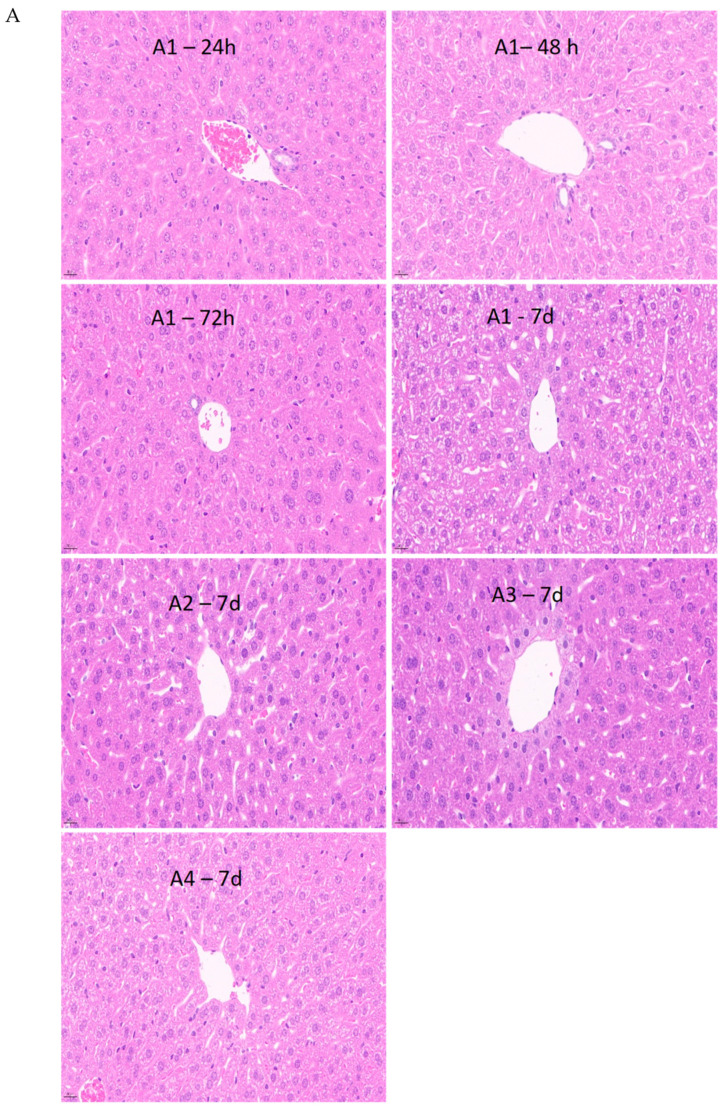

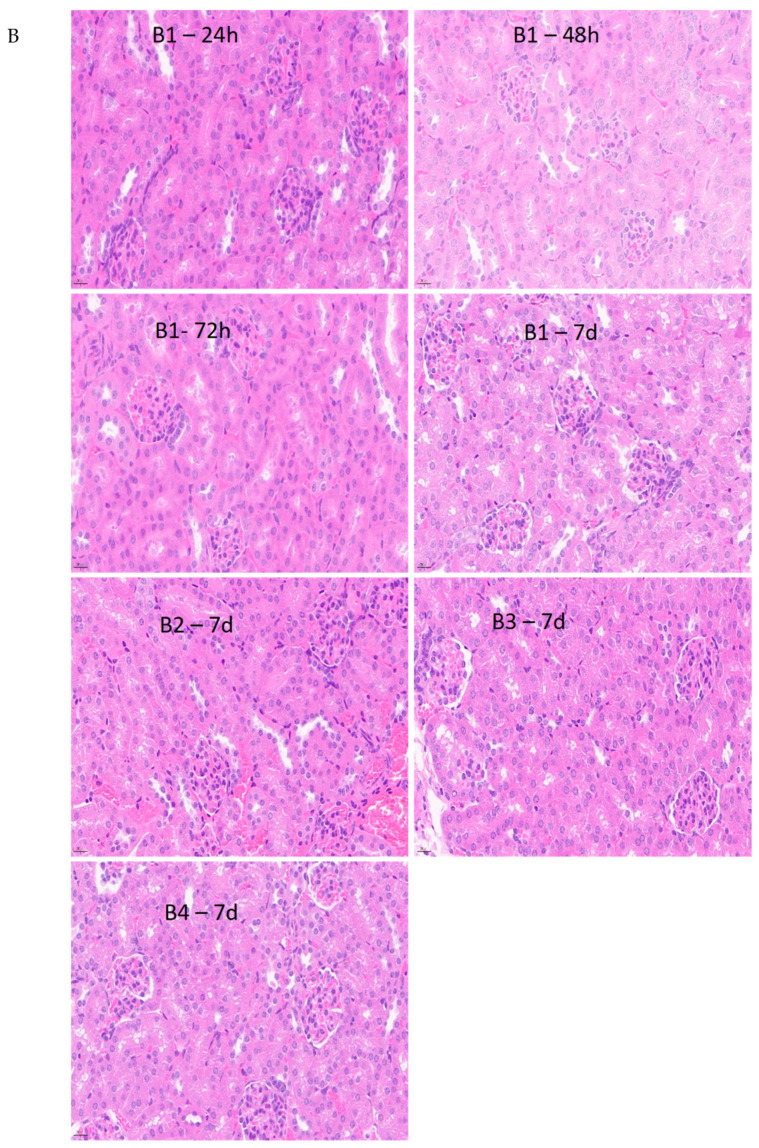
Histological examinations include liver (**A**) and kidney (**B**) sections. 1: MT—10,000 mg/kg-administered male mice; 2: male control; 3: MT—10,000 mg/kg-administered female mice; 4: female control. (Magnification: ×40). MT—Monterey almond hull powders.

Compared to the control group, the liver hepatic lobule, sinusoidal, plate, and hepatic cell structure were found to be normal in MT-10,000 mg/kg bw-treated groups both for female and male mice, as seen in Figure 6A. For all the sections harvested at 24 h, 48 h, 72 h, and 7 d, the structure of the kidney cortex and medulla of the treated mice were in a normal state compared to the control group, as shown in Figure 6B. It was demonstrated that there were no noticeable adverse effects of the MT-10,000 mg/kg bw administration on the mice liver and kidney system for acute toxicity studies. 

## 4. Discussion

Besides the previous usage of almond hull in the diets of livestock such as cows, sheep, hens, and pigs, almond hull has been reported for its various health-promoting potentials, like antioxidant, antidiabetic, and antihypertensive effects, using in vitro assays [22]. It has also been found that almond hull powder, with bioactive compounds and fiber, can reduce total cholesterol and triglycerides in hyperlipidemic male rats [17]. As almond kernels have been commonly consumed as food, Song et al. conducted a subchronic oral toxicity study of almond skins in rats, and the no-observed-adverse-effect level for almond skins was considered to be 10% (*w*/*w*) for both genders throughout the 90-day feeding study [23]. Even though almond hull has been utilized as a feedstock for decades, they are formally defined as a safe feed ingredient by the Association of American Feed Control Officials (AAFCOs), and the safety of almond hull as a livestock feed ingredient has been validated through the long history of cattle feeding and by many livestock feeding studies [13,14,15,24]. To fully utilize this agricultural byproduct and validate its safety, this study aimed to provide a detailed assessment of the toxicological characteristics of almond hull powders through acute oral toxicity experiments conducted on mice. Following the OECD 423 guidelines, a starting dose of 300 mg/kg bw was used when there was no prior information available about the substance, primarily for ethical reasons related to animal welfare. Notably, no instances of mortality or signs of toxicity were observed in this study. 

There was no significant alteration found in behavioral pattern and food intake throughout the 14 days of the acute oral toxicity evaluation, accompanied by the non-significant body weight variations. There was a significant difference in the initial weight of the BT-2000 mg/kg bw and 5000 mg/kg bw test groups, but there was no significant difference in the body weight of each group beyond this. Statistically, no significant variations were found in the organ to body weight index of the mice in all treatment groups, including all the vital organs of liver, kidney, heart, lungs, and spleen. The significant difference in the thymus gland in the BT and NP—300 mg/kg bw test groups was not considered as test-article-related; it could be due to inadequate exercises due to its small size. Similar phenomena have been reported by Pfeiffer, that for 6.5–9-week-old rats, the intrinsic and physiological factors greatly influence the thymus index [25]. 

In the acute oral toxicity evaluation of almond hull powders, various clinical biochemistry parameters have been introduced and assessed to explain the toxicity of the test subjects. It was suggested that the hepatotoxicity be monitored by quantitative analysis of the serum enzymes ALT, AST, and γ-GT and renal toxicity by urea and creatinine [26]. There were no significant changes in the liver and kidney profile tests of all the treatment groups, except for a variation found in ALT in the NP-300 mg/kg bw and 5000 mg/kg bw-treated groups. The blood levels of AST and ALT are known to significantly increase, potentially resulting from the destruction of liver cells in a toxic environment. Importantly, ALT has been commonly used as a more specific marker to quantify suspected liver cell damage due to their abundance in the cytoplasm of liver cells. The elevated values of ALT and AST may indicate liver injury [27]. However, the ALT ranges varied by the species of mice, and they were measured as 239.50 ± 141.20 UI/L for female BALB/c female mice, which is generally higher than BALB/c male mice at about 99.44 ± 39.61 UI/L [28]. Thus, the variations in ALT found in the test groups were suggested to be incidental, whereas the higher NP dosage at 5000 mg/kg bw showed a lower ALP than the mice dosed at 300 mg/kg bw, which was also later confirmed by the follow-up study on the hepatic and renal function using an additional control group, and a male mice group at a dosage of MT at 10,000 mg/kg bw. Furthermore, a high ALP level is often an indicator of biliary tract obstruction found in cholesterol liver disease [29], and a high level of total protein is associated with dehydration or increased synthesis by the liver [30], whereas there was only a statistically significant difference in ALP levels at 48 h (*p* < 0.01) and statistically significant differences in TP levels at 48 h (*p* < 0.01) and 72 h (*p* < 0.05) when compared to the male control group, suggesting non-test-article-related factors. Thus, the assessment of the biochemical parameters related to liver function exhibited reassuring outcomes, as all observed variations remained within the expected normal ranges for the mice under investigation in this study. 

Histopathological lesions were suggested to be correlated to the changes in the biomarkers in liver and kidney function in mice [31]. From the current study, the histopathological findings of the acute oral toxicity demonstrate no changes in kidney and liver in all the tested groups (Figure 5), as well as the male mice, administered with MT at 10,000 mg/kg bw with the time span from 24 h to 7 d (Figure 6A,B). Furthermore, there were no noticeable adverse effects of oral almond hull powder administration on the rat vital organ system, including liver, kidney, lungs, heart, and spleen at all the dosages tested for acute toxicity studies. 

To the best of our knowledge based on our review of the literature, this is the first time that the safety assessment of almond hulls using rodent models has been reported. These crucial findings from the current study are expected to lay a foundation for future research endeavors, particularly establishing suitable dosages in subsequent genotoxicity and sub-chronic toxicological studies. Furthermore, this study shall set the stage for in-depth explorations of mutagenic activity, reproductive effects, and the potential cholesterol-lowering properties of almond hull powders. Such comprehensive analyses will enrich our understanding of their safety and potential health benefits, supporting their safe utilization for human consumption.

## 5. Conclusions

From the above discussion, it is firmly concluded that almond hull powders sourced from the assessed varieties (BT, MT, and NP) exhibit unequivocal safety. This 14-day OECD 423 acute oral toxicity study solidly confirms the non-toxic or unclassified nature of these powders. This categorization is consistent with the hazard classification criteria stipulated by the Global Harmonized System (GHS). The absence of noteworthy deviations in functional and behavioral observations, along with the no mortality at the maximum administered dose of 5000 mg/kg bw for all three varieties (and 10,000 mg/kg bw for MT), underscores their safe nature.

## Figures and Tables

**Table 1:** Microbial, heavy metal, pesticide residue, and mycotoxins in MT almond hull powder samples.

	Result	MRL	Units	Method Reference
Microbial				
Mold (48 h)	<10		CFU/gram	AOAC RI PTM 051702
Yeast (48 h)	<10		CFU/gram	AOAC RI PTM 051702
*E. coli*	<10		CFU/gram	FDA BAM, Ch 4 (Plate)
Total Coliform	<10		CFU/gram	FDA BAM, Ch 4 (Plate)
*Salmonella*	Negative		/100 g	AOAC RI PTM #100701
Metals				
Mercury (DMA)	<4.00		ppb	EPA 7473
Arsenic (ICP-MS)	195		ppb	AOAC 993.14
Cadmium (ICP-MS)	16.2		ppb	AOAC 2015.06
Lead (ICP-MS)	271		ppb	AOAC 2015.06
Verified Residue				
Azoxystrobin	0.015	4	mg/kg	AOAC 2007.01
Methoxyfenozide	2.042	25	mg/kg	AOAC 2007.01
Chlorantraniliprole	1.484	5	mg/kg	AOAC 2007.01
Pyraclostrobin	0.334	7	mg/kg	AOAC 2007.01
Etoxazole	0.074	2	mg/kg	AOAC 2007.01
Spinetoram	Approaching LOD (0.010)	19	mg/kg	AOAC 2007.01
Fenpyroximate	0.028	3	mg/kg	AOAC 2007.01
Spirodiclofen	0.183	20	mg/kg	AOAC 2007.01
Hexythiazox	0.729	10	mg/kg	AOAC 2007.01
Bifenthrin	0.483	2	mg/kg	AOAC 2007.01
Cyhalothrin Lambda	Detected < LOQ (0.200)	1.5	mg/kg	AOAC 2007.01
Oxyfluorfen	0.081	0.1	mg/kg	AOAC 2007.01
Pendimethalin	0.694	6	mg/kg	AOAC 2007.01
Permethrin	Detected < LOQ (0.400)	20	mg/kg	AOAC 2007.01
Propargite	Approaching LOD (0.040)	55	mg/kg	AOAC 2007.01
Fenpropathrin	Detected < LOQ (0.100)	4.5	mg/kg	AOAC 2007.01
Tebuconazole	Approaching LOD (0.010)	6	mg/kg	AOAC 2007.01
Mycotoxins				
Aflatoxin B1	Not Detected		ppb	
Aflatoxin B2	Not Detected		ppb	
Aflatoxin G1	Not Detected		ppb	
Aflatoxin G1	Not Detected		ppb	
Total Aflatoxins	Not Detected		ppb	

**Table 2:** Effect of administration of almond hull powders on body weight indices in female mice.

Organs	BT (mg/kg bw)			MT (mg/kg bw)			NP (mg/kg bw)		
	300	2000	5000	300	2000	5000	300	2000	5000
Heart	0.443 ± 0.1	0.509 ± 0.058	0.513 ± 0.004	0.555 ± 0.023	0.518 ± 0.039	0.476 ± 0.055	0.549 ± 0.085	0.523 ± 0.019	0.525 ± 0.037
Liver	4.075 ± 0.164	3.878 ± 0.157	3.652 ± 0.126	3.77 ± 0.126	3.732 ± 0.143	3.632 ± 0.188	3.722 ± 0.078	3.995 ± 0.170	3.734 ± 0.045
Spleen	0.417 ± 0.009	0.424 ± 0.007	0.415 ± 0.037	0.429 ± 0.081	0.401 ± 0.026	0.409 ± 0.024	0.416 ± 0.021	0.424 ± 0.011	0.396 ± 0.036
Stomach	0.760 ± 0.11	1.109 ± 0.326	1.107 ± 0.208	0.896 ± 0.201	1.101 ± 0.201	0.866 ± 0.119	0.852 ± 0.022	1.044 ± 0.23	0.845 ± 0.064
Kidney	1.371 ± 0.043	1.356 ± 0.133	1.350 ± 0.107	1.414 ± 0.044	1.362 ± 0.056	1.276 ± 0.131	1.371 ± 0.086	1.411 ± 0.032	1.376 ± 0.009
Lung	0.729 ± 0.037	0.652 ± 0.037	0.637 ± 0.024	0.652 ± 0.061	0.629 ± 0.030	0.666 ± 0.052	0.623 ± 0.023	0.743 ± 0.137	0.604 ± 0.093
Thymus Gland	0.306 ± 0.071 *	0.261 ± 0.027	0.244 ± 0.03	0.203 ± 0.061	0.203 ± 0.009	0.210 ± 0.089	0.115 ± 0.062 *	0.172 ± 0.088	0.179 ± 0.064

All data are reported as the mean ± SD for n = 3 per group. Two-way ANOVA, followed by Tukey’s test. * Significantly different, *p* < 0.05. Organ to body weight index = (organ weight × 100)/body weight. Abbreviations of almond hull powder: BT—Butte, MT—Monterey, NP—Nonpareil.

**Table 3:** Effect of administration of almond hull powders on renal and liver function tests in female mice.

Parameters	Unit	BT (mg/kg bw)			MT (mg/kg bw)			NP (mg/kg bw)		
		300	2000	5000	300	2000	5000	300	2000	5000
CREA	µmol/L	33 ± 1.73	31.33 ± 2.52	32.67 ± 0.58	33.67 ± 2.08	32.67 ± 3.79	33.33 ± 4.73	35 ± 1	32.33 ± 1.15	36.5 ± 2.12
ALT	U/L	52.67 ± 7.51	63 ± 3	51.67 ± 6.11	61.67 ± 11.55	58 ± 2.65	60.67 ± 11.93	69.67 ± 6.11 *	50 ± 8.54	47 ± 1.41 *
AST	U/L	276.67 ± 117.01	191.33 ± 12.9	188 ± 37.47	209.33 ± 30.44	187 ± 12.29	236 ± 15.72	211.33 ± 10.97	181.67 ± 32.93	191.5 ± 6.36
ALB	g/L	21.03 ± 0.71	20.2 ± 0.4	21.43 ± 0.38	21.17 ± 0.5	21.53 ± 0.25	22.07 ± 0.45	21.63 ± 0.45	21.17 ± 1.19	21.75 ± 0.21
TP	g/L	59.33 ± 2.38	56.73 ± 0.5	60.53 ± 1.86	60.27 ± 2.65	60.8 ± 0.85	62.17 ± 1.64	61.7 ± 1.91	60.27 ± 3.01	61.1 ± 0.28
ALP	U/L	143.67 ± 23.35	144.33 ± 21.59	170.33 ± 5.77	150 ± 7.07	159.33 ± 4.04	183.67 ± 10.26	176 ± 12.53	158 ± 44.03	166 ± 19.8

All data are reported as the mean ± SD for n = 3 per group. Two-way ANOVA, followed by Tukey’s test. * Significantly different, *p* < 0.05. Abbreviations of almond hull powder: BT—Butte, MT—Monterey, NP—Nonpareil.

## Data Availability

Data available upon request due to ethical restrictions. The data presented in this study are available on request from the corresponding author following approval from the relevant ethical oversight bodies. The data are not publicly available due to ethical concerns.
